# Behavior of Composites Made of Quadriaxial Glass Fiber Fabrics and Epoxy Resin under Three-Point Bending

**DOI:** 10.3390/polym16131925

**Published:** 2024-07-05

**Authors:** Ioana Gabriela Chiracu, George Ghiocel Ojoc, George Cătălin Cristea, Mihail Boțan, Alina Cantaragiu Ceoromila, Cătălin Pîrvu, Alexandru Viorel Vasiliu, Lorena Deleanu

**Affiliations:** 1 Department of Mechanical Engineering, Faculty of Engineering, “Dunarea de Jos” University, 111 Domneasca, 800201 Galati, Romania; ioana.chiracu@ugal.ro (I.G.C.); george.ojoc@ugal.ro (G.G.O.); alina.cantaragiu@ugal.ro (A.C.C.); alexandru.vasiliu@ugal.ro (A.V.V.); 2 Autonomous Flight Technologies, 1 Aeroportului, 077060 Clinceni, Romania; 3 National Institute for Aero-Space Research (INCAS) “Elie Carafoli”, 220 Iuliu Maniu, 061126 Bucharest, Romania; cristea.george@incas.ro (G.C.C.); botan.mihail@incas.ro (M.B.); pirvu.catalin@incas.ro (C.P.)

**Keywords:** quadriaxial glass fiber fabric, composite, epoxy resin, three-point bending test, test rate, Young’s modulus, flexural stress at first peak, flexural strain at first peak, energy at first peak, force at first peak

## Abstract

This paper presents experimental results from three-point bending tests for a composite made of quadriaxial glass fiber fabrics and an epoxy resin. Two composites were tested, one with 8 layers and the other with 16 layers; both had the same matrix (the epoxy resin). Tests were carried out, using five different test rates from 10 mm/min to 1000 mm/min. The following parameters were recorded and calculated: Young’s modulus, flexural stress, flexural strain, energy, force, and all four for the first peak. The experimental data reveal no sensitivity for these materials based on the test rates, at least for the analyzed range; but, the characteristics for the thicker composite, with 16 layers of fabric, are slightly lower than those for the thinner composite, with 8 layers. The results pointed out that, for the same thickness of composite, certain characteristics, such as stress at the first peak, the flexural modulus, strain at the first peak, and energy at the first peak, are not sensitive to the test rate in the range 10–1000 mm/min. The energy at the first peak is double for the 16-layer composite compared to the 8-layer composite, but the specific energy (as energy on cross-sectional area) has close values: 103.47 kJ/m^2^ for the 8-layer composite and 106.51 kJ/m^2^ for the 16-layer composite. The results recommend this composite for applications in components with resistance to bending or for low-velocity impact protection.

## 1. Introduction

Composites have been gaining a greater share among materials used in industry, transport, and agriculture because research has been focused on pointing out their particular characteristics [1] and on increasing their reliability and durability [2] in design solutions [3], especially for the aircraft, marine, and military industries [4,5,6].

Testing composites is a vital stage in introducing them in actual systems. Even if modeling and simulation help with evaluating the load and durability of composites, experimental work is commendatory for a reliable design [7,8]. The behavior of composites in actual applications is controlled by many factors and these should be taken into consideration throughout the testing; from laboratory-scale tests, which help with revealing failure mechanisms, to large, actual-scale tests, as shape and dimensions influence the response of the structures tremendously in terms of actual functioning [9,10,11,12].

Among the other mechanical tests for composites, three-point bending tests are essential for assessing the mechanical properties of composite materials [13,14,15]. The importance of this test will be argued here. This test is useful for mechanical properties’ evaluation as it provides crucial data regarding the stiffness, strength, and ductility of composite materials. By subjecting the composite to bend loading, engineers can evaluate its behavior under different loads and geometries; therefore, they determine its suitability for particular applications.

Many structural components in engineering applications, such as beams and panels, use very different materials [16,17], but are subjected to bending loads during their service life. Conducting three-point bending tests helps engineers to understand how composites will perform in these actual scenarios, enabling them to design and optimize composite structures accordingly.

The behavior of composites under bending loads can reveal important information about their internal structure and properties regarding influencing factors, such as fiber orientation, matrix stiffness, and interfacial bonding [18,19,20]. This characterization is vital for understanding the composite’s overall performance and for quality control during fabrication and for functioning monitoring and failure evaluation [21,22,23].

These tests are commonly used as a quality assurance tool for composite materials. By comparing the results of bending tests using established standards [24,25] and specifications, manufacturers can ensure that their composites meet the required mechanical properties and performance criteria. Also, a new composite could be compared to older solutions in terms of bending characteristics.

The obtained data can be used to optimize the design of composite structures for specific applications. Engineers can adjust factors, such as fiber orientation and volume, stacking sequence, and matrix composition to enhance the material’s bending characteristics.

In the event of a structural failure or performance issues, three-point bending tests can help engineers analyze the cause of failure and identify solutions for improving the composite or the design. Three-point bending tests are also a valuable tool for researchers and designers in developing new composites or manufacturing processes. By systematically studying the bending behavior of different formulations or processing techniques, they can innovate, advance, and improve the family of composite materials.

The earliest documented three-point bending tests on composites may be traced back to the mid-20th century; they likely occurred sometimes in the 1950s or 1960s. Composites, as they are understood today, have been gaining attention and being systematically studied. One of the pioneers in composite materials’ research was Norman de Bruyne [26], who conducted seminal research work in the field.

These early tests would have been conducted using basic equipment, possibly to be adapted for this purpose. The three-point bending test involves applying a load to a sample placed on two supports with a third point applying force to the center. The aim is to measure the flexural properties of the material, such as modulus of elasticity, flexural stress, and strain at failure. Failure has to be detailed for a composite class, as the complete break of the tested specimen is rare. Nowadays, there are standardized tests for assessing the quality of such materials [24,25]. Using specimen geometry, the test procedure and even methods to obtain the specimens from the standards make comparison easier among a very diverse variety of composites. These tests laid the groundwork for more sophisticated testing techniques and contributed significantly to today’s understanding of composite materials’ mechanical behavior.

Fan W. et al. [27] studied the behavior of hybrid composites, specifically those with carbon and glass fibers, in terms of bending using the three-point test, both statically and in fatigue. The quasi-static tests were done with a rate of 2 mm/min, the span length being 64 mm. The test was repeated three times for each formulated composite and the failure particularities were pointed out by scanning electron microscopy.

In 2022, Jeon et al. [28] studied the flexural behavior of two different materials, steel and aluminum alloy, bonded with an adhesive that exhibits different adhesion strengths, from 22 to 30 MPa, and reported several characteristics that are also analyzed in this paper. The conclusion could be qualitatively related to glass fiber composites: the bending stress increased with the adhesion strength, meaning that matrix quality to adhere to the reinforcement has a high influence on bending behavior.

Nazaripoor et al. [29] tested two sets of random short glass-fiber-reinforced polyester composite, with a fiber volume fraction of 12.5%, and 15%, respectively. The test rate was very small (1.0 mm/min), with two different span lengths (177.8 mm and 264.2 mm). The mechanical strength increased from the lower to the higher fiber volume fraction, and so did the maximum strain (~17%), maximum flexural stress (~22%), and flexural chord modulus (~8%). But, with the two different thicknesses, 4.6 mm for 12%V glass fibers and 8.7 mm for 15%V glass fibers, the results were influenced by this combination (thickness, glass fibers concentration) and not only by the increased content of glass fibers.

Petrescu et al. [30] studied the influence of span length for a type of carbon fiber composites, considering a step of 10 mm, for a span length between 60 mm and 100 mm on the load–displacement curve and on Young’s modulus values.

Even if the three-point and four-point bending tests are the subject of an international standard, for materials with different structures [24], research data are reported for very different specimens, made of very different materials, with different shapes and dimensions because of specific applications the materials are intended to be used [15,31].

Phunpeng et al. [32] tested composites with carbon fibers in an epoxy matrix, considering the flexural strength as output parameter, input parameters being ply orientation, manufacturing, width, thickness, and graphite filler percentage. A plain-woven carbon fiber fabric, with a weight of 200 g/m^2^ and a density of 1.8 g/cm^3^, an epoxy resin ER550, and a graphite filler (with a particle size of 5 μm) were used for the composite fabrication. The test rate was 5 mm/min, and the span length was 100 mm. The specimens had dimensions of 191 mm × 20 mm × 2 mm. The experimental results help the authors predict the composite behavior using an artificial neural network, but tests with different test rates could be useful for designing applications with these composites.

Ma et al. [33] investigated the behavior in flexural and tensile tests of two types of composites, both have the same carbon fibers, but one type used thermoplastic resin Nylon 6 as matrix and the other one—a thermosetting epoxy resin. The thermoplastic resin causes the composites to have uneven fiber distribution and higher void content, but the epoxy resin, at similar fiber volume, means that the composites have almost-uniform fiber distribution and a lower void volume. Tensile and bending tests for 90° CF/Epoxy laminates recorded almost double values tensile and flexure stress at break as compared to the composite with Nylon 6 matrix with similar fiber volume fraction.

Turaka et al. [34] reported the influence of glass fiber orientation (0°, 90°, 0°/90°, and ±45°) in an epoxy matrix (tensile limit 100 MPa, tensile modulus 7.94 GPa), with 12 layers. For flexural tests, the following characteristics were obtained: flexural stress 138.4 ± 6.44 MPa for 0° and 77.26 ± 5.83 MPa for combination 0°/90°. Test were performed with a test rate of 1 mm/min and a span-to-thickness ratio of 16. It was interesting to notice that flexural stress has the same slope for these two mentioned orientations till around 75 MPa.

Monjon et al. [35] obtained for a glass fiber composite with 63%wt fiber (eight layers of woven bidirectional fabric, with 195 g/m^2^), 632.5 MPa as flexural stress, and 22.1 GPa for the flexural modulus; they also tested a hybrid composite with glass fibers, carbon fibers, and aramid fibers. The high value for flexural stress and modulus could be generated by the high fiber content and high quality of the resin. They produced the composites, simple or hybrid, with the same resin, with an Ebalta AH 150 resin and IP 430 hardener.

The glass fiber composites formulated by Birleanu et al. [36] had six layers of 270 g/m^2^ glass fiber twill fabric and a bicomponent epoxy resin (Epikotetm Resin MGS LR 135 and Epikuretm Curing Agent MGS LH136, with a mass ratio of 100: 35.2, supplied by HEXION GmbH, Duisburg, Germany), resulting weight fraction ratios in the range 54–68.5% for composites with 2 mm thickness. Flexural tests were performed for 2 mm/min and for the lowest fiber fraction there was obtained the higher value of flexural stress (464.8 ± 11.5 MPa). The lowest (415.5 ± 21.5 MPa) was recorded for the maximum value for fiber fraction. Also, flexural strain decreased with the weight fraction increase, the modulus being severely reduced for the lowest value of weight fraction (15 GPa). But tensile stress increases with the weight fraction increase. These tests pointed out that composites have a particular behavior depending on the loading type (bending or traction, in that study).

This paper analyzes the behavior of high-quality composite made of quadriaxial glass fiber fabrics and an epoxy resin, under three-point bending, for different rate tests. There was pointed out the influence of test rate and composite thickness on flexural characteristics.

## 2. Materials and Methods

The fabric used in this research is layered in four substrates with orientation (0°/+45°/90°/−45°), which assumes that the fabric will have a quasi-isotropic behavior. The trade name is 1200 g/m2 Quatriaxial Glass Cloth (0°/+45°/90°/−45°) 127, with code WTVQX1200-1 E-glass, Q1200E10Q, and was supplied by Castro Composites (Pontevedra, Spain) [37]. The fabric architecture consists of four layers with different orientations, namely 0°, 45°, 90°, and 45°. Each sublayer has its own areal density (sublayer 1—283 g/m^2^; sublayer 2—300 g/m^2^; sublayer 3—307 g/m^2^; sublayer 4—300 g/m^2^) and the stich adds 10 g/m^2^, resulting a total areal density of 1200 g/m^2^ (± 3%).

**Figure 1:**
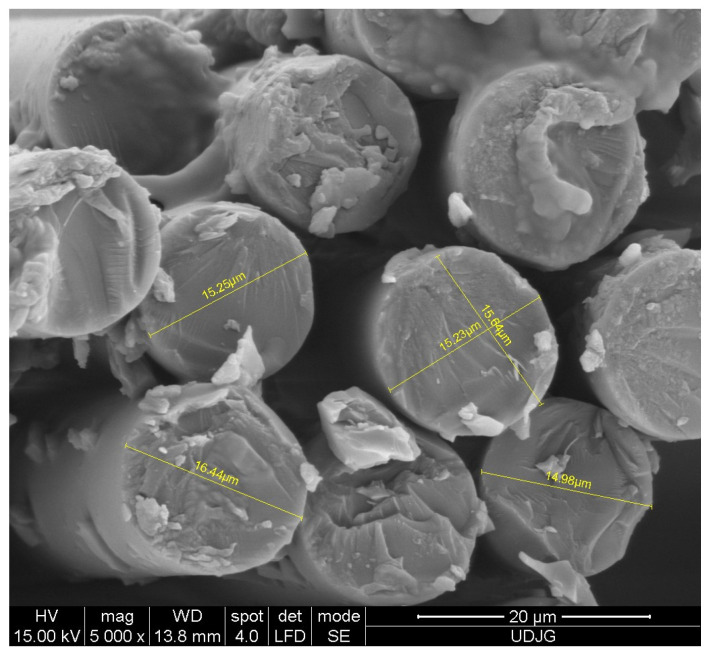
Measurements of glass fiber diameters, as obtained by cutting the fabric with a dedicated scissors [38].

Figure 1 shows the size of the glass fibers, measured by the help of the scanning electron microscope. The width of a thread is ~3 mm, the thickness being ~180 μm. The diameters of the glass fibers are approximately ~15–18 μm. The glass fiber rolls were stored in the laboratory at a relative humidity of 45–60% and a temperature of 18–30 °C, as recommended by the manufacturer. The same fabric was used in [38,39].

Velmurugan et al. [40] present bending test results for hybrid composites made of glass fiber twill fabrics and sisal fibers, but the authors of this study were interested in the simple glass fiber composite, as it was formed with an epoxy resin that has 23 MPa tensile limit and a Young modulus of about 8 GPa; the composite made of glass fibers and this resin had a flexural limit of about 210 MPa and a flexural modulus of 12 GPa, as found by a three-point bending test with a span–depth ratio of 16:1 and a span length of 70 mm, with a test rate of 5 mm/min. It is difficult to compare results even for the same test, but results from [40] and the results presented in this study point out the importance of the epoxy matrix on bending characteristics. Another factor influencing the bending results is the fiber fraction that is not given.

**Figure 2:**
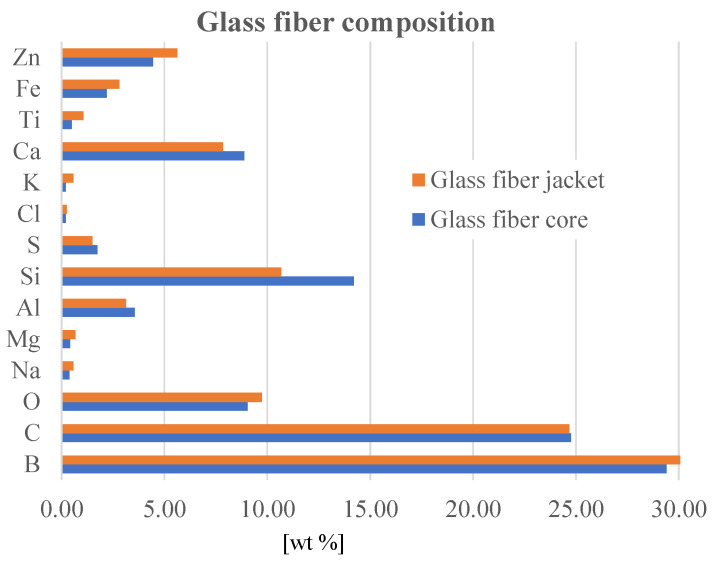
Glass fiber elemental composition.

Figure 2 presents the average elemental composition of the glass fibers. The surface and cross-section morphology and fiber–glass fabric characteristics were analyzed through the scanning electron microscopy (SEM) technique. Imagistic results were acquired by means of SEM microscope model Quanta 200 from FEI (Thermo Fisher Scientific, Hillsboro, OR, USA), operating at 15–19 kV accelerating voltage, in low-vacuum state. Electron secondary imaging allows to capture both macro- and micro-scale surface details, relevant for this study. SEM apparatus is equipped with an energy-dispersive X-ray (EDX) analyzer from AMETEK Limited (Leicester, UK), used to determine the semi-quantitative elemental composition of the sampled volume (in cross-section). EDX detector is made of lithium-drifted silicon, solid-state device. A pre-condition to SEM-EDX analysis consists of a thin metal (here, gold) layer sputtering (Sputter Coater equipment from SPI Supplies, West Chester, PA, USA) to increase sample conductivity and to enhance image resolution.

Nine measurements for the core (cross-section) and four measurements for the fiber jacket have been carried out. It can be seen that boron (B), carbon (C), aluminum (Al), silicon (Si), and calcium (Ca) predominate, with traces of iron (Fe), zinc (Zn), and titanium (Ti), the particulars of the composition being assigned to the extraction area of the raw material.

Epoxy resins are widely used as a matrix in strengthening composites, with applications in the aerospace industry, the marine industry, and ballistic protection.

It was selected Biresin^®^ CR82 two-component resin with CH80-2 hardener (coded B) [41]. The mixing ratio resin (A)/hardener B is 100/27 (produced by Sika Deutschland GmbH Subsidiary, Bad Urach, Germany). The minimum shelf life for Biresin^®^ CR82 (A) resin is 24 months and Biresin^®^ CH80-2 hardener (B) can be stored for 12 months at room temperature (18–25 °C) if the original container is not opened. If stored at a lower temperature, the crystallization of resin A may occur but this can be avoided by keeping the temperature at least 60 °C for a sufficient time. Density is 1.11 g/mL for component A and 0.99 g/mL for component B. The potlife of the hardener is approx. 80 min. Mechanical characteristics of resin Biresin^®^ CR82 and hardener Biresin^®^ CH80-2 are given in [39], following the procedure in ISO 527-1 [42]: a tensile strength of 85 MPa, a tensile modulus of 3.25 GPa, a strain at break of 5%, a flexural limit of 125 MPa, a flexural modulus of 3.2 GPa, a compression limit of 107 MPa, and an impact resistance, as in ISO 179-2 [43], of 21 kJ/m^2^.

On the active zones of the press, an extraction wax CIREX CP 10 (supplier Airétec, distributor RomPolimer Composites, Bucharest, Romania), used for epoxy resins, was ap-plied with a brush or sponge in one or two coats; there was a wait for the solvents to evaporate before applying the second coat or contacting the laminate. The first layer of fabric (having 300 mm × 300 mm as area) was put into the press, layered up with resin (using a brush), and then the second layer was added and layered up. The operation was repeated till the desired number of layers were covered with resin. A simple plate of wood, with the applied wax, was put on top of the composite and the press was activated with the help of the jack. Small markers were put on the side of the press to maintain the composite thickness constant. The panels were kept at least 8 h in the press. The authors applied the following heat treatment to the fabricated plates: naturally aged for 7 days, at 20–23 °C, then holding at 60 °C for 6 h in an oven and cooling in laboratory atmosphere.

Composite manufacturing has particular characteristics, its advantage being that it eliminates the severe conditions specific to other fabrication processes of components with similar purpose (very high melting temperatures, for ceramics and metal alloys, high pressures for shape modification, further processing to achieve the desired shape, such as cutting and grinding, molding, additive processes, etc.) [44,45,46,47,48].

After the natural ageing and after applying the heat treatment, the samples for three-point bending were cut with the help of the high-speed disk (dry regime). The samples dimensions were 160 mm × 14 mm. The tests carried out for this study were cut from two plates of the same thickness; the information of these plates is given in Table 1.

**Figure 3:**
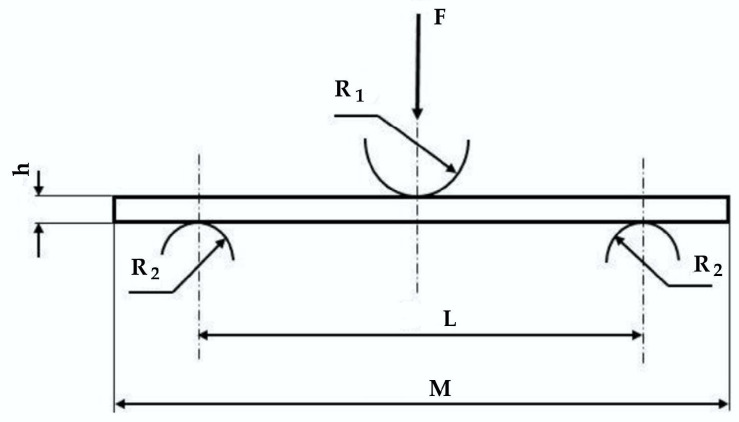
The sketch of the three-point bending test.

The test specimen and the main dimensions are given in Figure 3, F is the applied force, R_1_ is radius of loading roller, R_2_ is the fixed support radius (R_1_ = R_2_ = 5 mm), h is the specimen thickness. L = 100 mm is the span length between supports and M = 160 mm and is the specimen length, b is the width of the specimen. Each set of test conditions is characterized by the test rate and the number of layers of the composite. Eight-layer and sixteen-layer samples of composite were tested. Tests with five different test rates were performed: 10 mm/min, 100 mm/min, 200 mm/min, 500 mm/min, and 1000 mm/min. Five tests for each composite and test rate were carried out, except for the composite with 8 layers that has only four tests for 100 mm/min and 1000 mm/min. For each sample, width and thickness were measured with a high-precision vernier.

Tests were performed on a device for three-point bending, the Instron 5982 mechanical test facility, equipped with a 100 kN force cell, with dedicated software (from INCAS, Bucharest, Romania), and a load measurement accuracy to +/−0.5% of reading down to 1/1000 of load cell capacity.

## 3. Results and Discussion

For each test, the following characteristics are calculated:

- The flexural stress produced by the maximum value of the recorded force, F_max_,
  σ_max_ = 3 × F_max_ × L/(2 × b × h^2^) (1)
  where L is the span length (kept 100 mm for all tests), b is the width of the specimen, and h is the thickness of the same specimen.
- The flexural strain for the same force,
  ε_max_ = 6 × d_(Fmax)_ × h/L^2^ (2)
  where d_(Fmax)_ is the displacement reached when F = F_max_.
- The energy absorbed by the composite till the load reaches F_max_, d_(Fmax)_ being the displacement reaches when F = F_max_
  E_(Fmax)_ = 0.5F_max_ × d_(Fmax)_ (3)

This formula of energy approximation was used taking into account that the force–displacement curve has a large curvature radius and this calculation gives a lower value as the recorded one, but the values are close enough to safely cover the design:

- Absorbed energy per cross-surface unit (also named specific energy), E_s_, calculated for F_max_ and initial cross-section area of the specimen, A_0_ = h × b; h is the average thickness and b is the average width of the specimens tested under the same conditions (number of layers and test rate).

E_s(Fmax)_ = E_(Fmax)_/(h × b) (4)

The time necessary to reach F_max_ was also analyzed, noted with t_(Fmax)_.

A difference between the values calculated with Formula (3) and the recorded values for this characteristic was noticed, as the dedicated software of the testing machine calculated the entire area under the force–displacement curve (also noted F-d), E_(Fmax)_ being a triangular approximation of this area.

**Figure 4:**
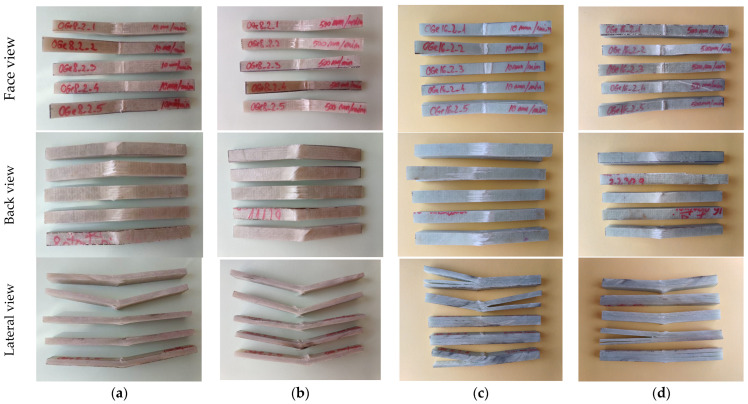
Set of five samples of composite with 8 layers, tested at different test rates: (**a**) 8-layer composite, 10 mm/min; (**b**) 8-layer composite, 500 mm/min; (**c**) 16-layer composite, 10 mm/min; (**d**) 16-layer composite, 500 mm/min.

Figure 4 presents the set of five samples of composite samples with 8 layers of quadriaxial fabric and the 16-layer composite samples, used for bending tests. Qualitatively, the failed samples are resembling for the same thickness. These photos, taken at macro level, point out failure zones: the contact zone with the loading roller; the zone near this one, with the first layer (s) crushed in compression; the bottom zone under the roller action direction, with tensile breakage of yarns and fibers. Delaminations are visible in the lateral-side views and the differences between the thin and thicker composites are pointed out. It is obvious that the thicker composite fails under a much lower displacement of the roller. Qualitatively, there are differences in damage aspects for the same composite, but these are tested at different test rates and also between the two composites tested at the same rate. For the eight-layer composite, the face damage to the first layer includes the compressive, crushed zone under the roller and the breakage of the first layer near the roller. The damaged zones seem to be larger for the higher test rate. The thicker composite presents larger de-bonding on the last layer (discolored zones) as compared to those of the thinner composite. The lateral view reveals delamination, a crush zone under the roller, and the breakage of the first layer because of the tensile bending action induced by the roller displacement. For the thicker composite, the delamination is severe; it is propagated up to the edge of the samples.

**Figure 5:**
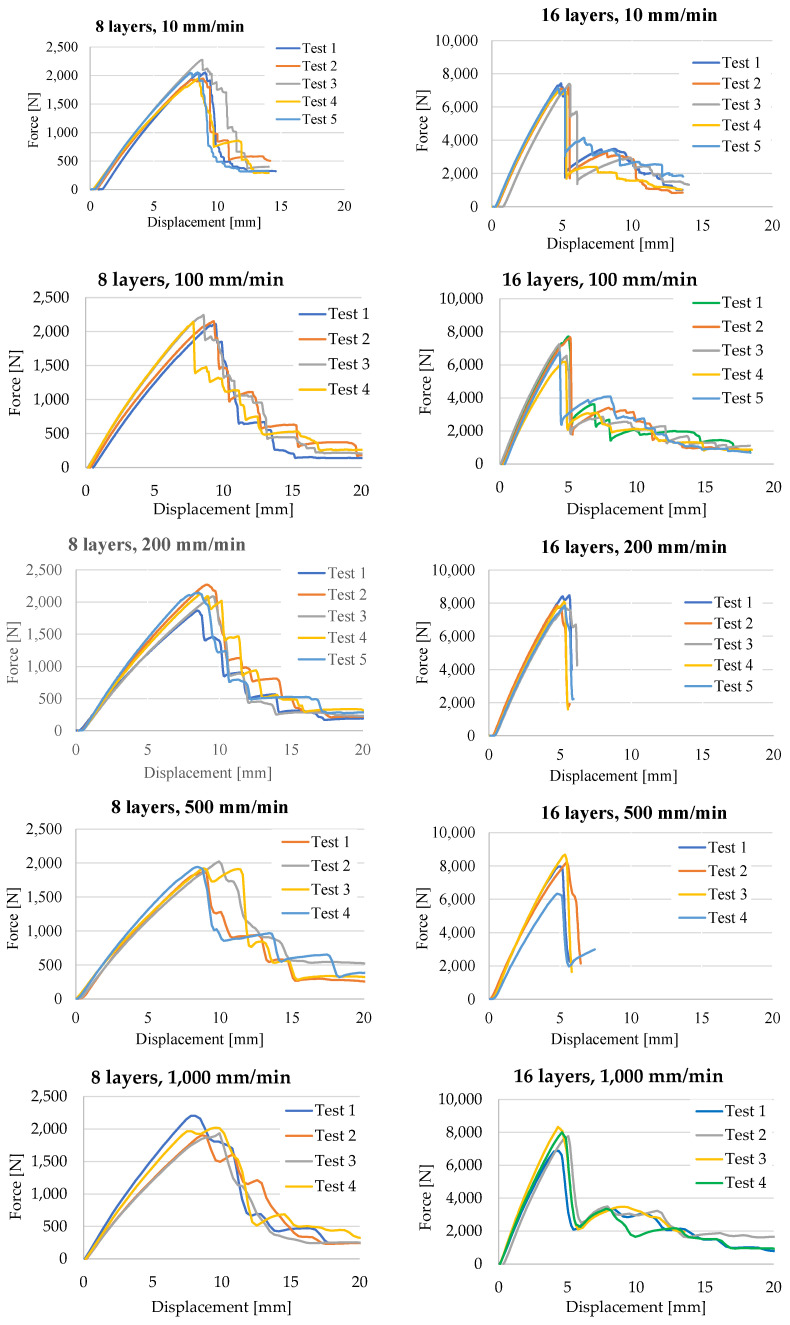
Force–displacement curves for the three-point bending tests.

Figure 5 presents the force–displacement plot for all tests included in this study. For each composite thickness and test rate, the number of tests is given in the legend.

Analyzing the force–displacement curves, one may notice that, up to F_max_ (the maximum recorded force, named force at first peak), the curves are similar in shape and the spread space (band) is small; but, after reaching this value, the curves evolve differently, on larger bands, and the shape is only qualitatively similar. This is why the designer tries to use a safe portion of the force–displacement curve, with an adequate safety coefficient.

The scale of the F axis was set at 2500 N for the composite with 8 layers and at 10,000 N for the 16-layer composite. The scale for the X axis (displacement) was the same for the same test rate. Qualitatively, the following conclusions can be drawn comparing the curves.

- F_max_ is in the range 1800–2300 N, for the thinner composite and 7800–8300 N for the thicker one, meaning F_max_ is proportional to the composite thickness.
- The same tendency was noticed for the displacement.
- The time till the maximum value of the bending force decreases drastically from the values characterizing the slowest tests (10 mm/min): F_max_ occurs around the moment of 51 s from loading, for the thinner composite and at 29.6 s for the thicker one; then, this decrease continues depending on the test rate but not so rapidly.
- The slopes of the curves till F_max_ are continuous and in a narrow band; thus, designers could rely on them (using an average value for the flexural modulus).

**Figure 6:**
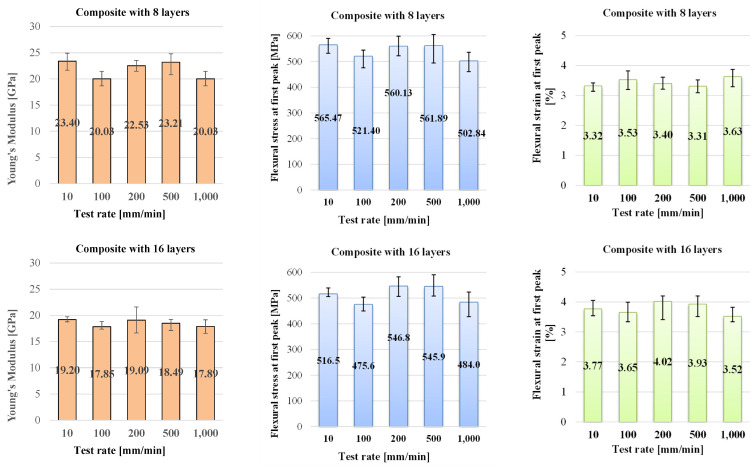
Mechanical characteristics for the tested composites.

Figure 6 presents the average values for each characteristic and their spread ranges, as recorded or calculated. Flexural modulus has average values between 19.6 GPa and 23.4 GPa for the eight-layer composite and lower ones for the thicker composite, between 17.8 GPa and 19.2 GPa, meaning this characteristic becomes lower when the thickness of the composite increases. Flexural stress at peak force, F_max_, calculated with relation (1), has the same tendency. The highest value for the eight-layer composite was 565.47 MPa (obtained for the slowest test rate of 10 mm/min) and the minimum value was 502.8 MPa, the difference representing only 11% of the higher value.

For the 16-layer composite, the extreme values are lower, 516.5 MPa and 478.8 MPa, with the same percentage being lower at 7.29%. These values are higher than other values found in open-access literature, being argued by the high quality of the epoxy matrix, the well-controlled laboratory technology, and the high mass ratio of fibers in the composites (0.74–0.75). These are several values of flexural stress for other glass fiber composites, with notices on the results:

- A flexural limit of 210 MPa and a flexural modulus of 12 GPa, but an epoxy resin with 23 MPa for tensile limit and a flexural modulus of about 8 GPa, tested at 5 mm/min using a three-point bending test [40].
- Values of 396.1 ± 24.1 MPa in the tensile tests at 5 mm/min, for a composite with 1.68–1.98 mm as the thickness [49].
- A value of 381.62 MPa for a hybrid composite of glass fiber and aramid fiber, with a thickness of 4 mm, and using an epoxy resin, LY 5052 [50].
- Values of 316.13 MPa and 23.22 GPa for the flexural modulus for a glass fiber composite with seven layers (each layer has an areal density of 360 g/m^2^), with a 3 mm thickness; the weight ratio of epoxy to fibers was 50:50; the epoxy polymer was Lapox L-12, and the hardener was K-6; the test rate was 5 mm/min [51].

The flexural strain is also insensitive to test rate, the values being in the range of 3.32–3.57% for 8-layer composite and 3.77–3.49% for 16-layer composite.

**Figure 7:**
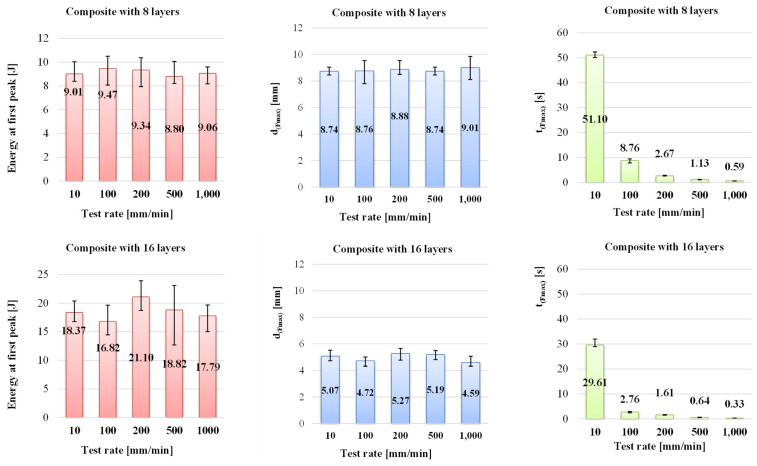
Energy at the first peak, displacement at F_max_, noted d_(Fmax),_ and the time till F_max_, noted t_(Fmax),_ for the tested composites.

Figure 7 presents the three other characteristics of the bending tests, namely the energy at the first peak, the displacement at F_max_, noted as d_(Fmax)_, and the time at F_max_, t_(Fmax)_. Comparing the plots for the energy at the first peak of the force, it is obvious the insensitivity of energy at the first peak and the displacement d_(Fmax)_ on the test rate, at least for the range of 10–1000 mm/min. These are sensitive to the composite thickness (or number of layers). Thus, the energy at the first peak is almost double if the composite doubles its layers. The displacement d_(Fmax)_ is reduced by half when the composite has 16 layers as compared to that for 8-layer composite.

For the specific energy, if it is calculated as the absorbed energy per cross-section area of the specimen, with relation (4), one may notice that values are very close for both composites (Table 2). Each value for the energy at the peak, h, b, and the specific energy, are the averages of the values obtained from the tests with the same test rates and composite thicknesses.

Analyzing the values for energy at the first peak, the composite with 8 layers of quadriaxial glass fibers has the values in a narrow range, but for the thicker composite (16 layers), the values are spread on larger range. The energy at the first peak has a double value as compared to that characterizing the thinner composite. And the ratio 2:1 is the same to the layer ratio. In order to validate this observation, it would be recommended to extend the study to other composites made with different numbers of layers. The same conclusion is drawn for the specific energy.

**Figure 8:**
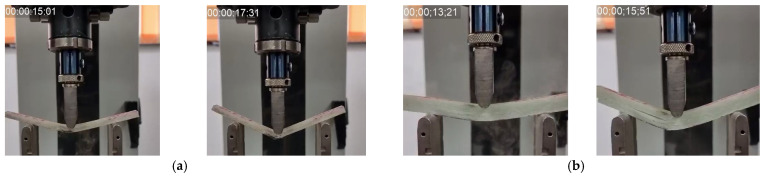
Successive images of the three-point bending test for two specimens of different thicknesses, at 200 mm/min test rate: (**a**) 8-layer composite and (**b**) 16-layer composite.

Figure 8 presents successive moment of two tests at the same test rate in order to point out, qualitatively, the difference in duration until the composite fails.

In materials science and engineering, composites tested in three-point bending with properties that do not depend on test rate typically have properties that are predominantly controlled by the matrix material rather than the reinforcement. Here are a few common composites, some of their properties being relatively insensitive to a certain test rate range, especially for elastic characteristics:

- Fiber-reinforced polymers [52,53,54];
- Metal matrix composites [55].

In these composites, the properties that are less sensitive to test rate are typically those related to the matrix material, such as its elastic modulus, yield strength, and fracture toughness. Reinforcement materials may contribute to specific mechanical properties, but their influence on overall behavior in three-point bending tests may be less pronounced compared to the matrix.

In composites, epoxy resins often exhibit consistent mechanical properties across a range of test rates, especially when reinforced with fibers such as carbon or glass. The properties of the epoxy matrix, such as its stiffness and toughness, typically dominate the behavior of the composite in three-point bending tests.

Papanicolaou, G.C. et al. [56] reported that, except for a small range with a very low test rate from 0.5 mm/min to 10 mm/min, the epoxy resin RenLam CY219 (Bisphenol A) combined with a curing agent HY 5161 (Diamine) of a ratio 2:1 by weight had a constant Young’s modulus in the three-point bending test of 2.5 GPa. Specimens used for the flexural tests had dimensions of 100 mm × 12.8 mm × 2.5 mm and a span length of 63 mm.

Ceramic matrix composites reinforced with fibers, such as silicon carbide, can possess properties that are less sensitive to test rate, particularly in high-temperature applications. The properties of the ceramic matrix, such as its high temperature stability and fracture toughness, are primary factors in determining the behavior of the composite under bending loads. But, at room temperature, they are sensitive to the strain rate [57,58].

Righi R. [14] tested composites with carbon fibers in an epoxy resin, for 3.3 × 10^−^^5^ m/s to 10 m/s, and pointed out that, for thin plates (1.5 mm), the sensitivity of the composite to the test rate was low. Zhu et al. [59] tested the glass fiber composite with an epoxy resin as the matrix, the thickness being 15 mm, close to 12–13 mm of the tested composite for this study, but only for 10 mm/min.

Reis et al. [60] performed three-point bending tests, using specimens made of unidirectional carbon fibers and different tapes made of polypropylene and modified PP; the technology implies hot pressing of PP tapes and carbon fibers (the fiber volume fraction in the hybrid composites being around 9.3%). The samples measure 40 mm × 10 mm × 0.85 mm and the span length was 20 mm. The range of test rate was 0.2 mm/min up to 200 mm/min. The researchers reported a very high similarity of slopes (of Young’s modulus) for all tested rates, but different maximum stress at break; this value increased with the test rate increase.

This insensitivity to the test rate for the particular composites has been reported by Kawata et al. [61], even from 1981, for carbon/epoxy specimens, with a plain-woven cloth reinforcement.

The conclusion of this discussion on test rate influence of three-point bending characteristics is that experimental reports reveal particular behavior under bending.

Thus, this type of composite, tested for this study, exhibited no clear sensitivity with test rate, for each thickness (or number of layers). The values for Young’s modulus flexural stress at first peak for the thicker composite are lower. The strain at first peak is a little bit higher than the thinner specimens; the displacement at the maximum value of the recorded force is lower for the thicker specimens. From these six characteristics analyzed, only t_(Fmax)_ has a dependence on test rate, decreasing qualitatively with the increase in test rate, as a power function. The energy at first peak depends on thickness, being almost double that the energy absorbed by the thinner specimen.

**Figure 9:**
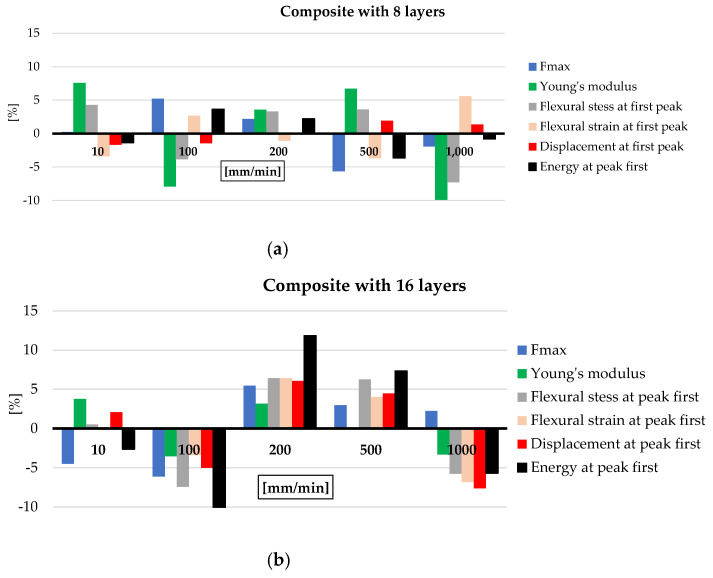
Percentage difference of each value obtained at a particular test rate calculated to the average values of each characteristic for all test rates.

Figure 9 presents the percentage differences of the flexural characteristics for those that exhibit no clear sensitivity on test rates: (a) for the composites with 8 layers and (b) for composites with 16 layers. The calculation will be explained only for F_max_ and for the composite with eight layers, but the same calculation procedure will be kept for all other parameters in Figure 9. The zero line represent the average value obtained with the values characterizing each test rate:

F_max av_ = (F_max(10mm/min)_ + F_max(100mm/min)_ + F_max(200mm/min)_ + F_max(500mm/min)_ + F_max(1000mm/min)_)/5

The average values of F_max_, for each test rate, are F_max(10mm/min)_ = 2059.86 N, F_max(100mm/min)_ = 2162.76 N, F_max(200mm/min)_ = 2100.07 N, F_max(500mm/min)_ = 1939.50 N, and F_max(1000mm/min)_ = 2015.83 N, resulting in F_max av_ = 2055.60 N.

The percentage difference between F_max av_ and each average value for a certain test rate, ΔF_max(x)_ is calculated as

ΔF_max(y)_ = [(F_max av_ − F_max(y)_)/F_max av_] × 100 [%] (5)

where y is taken from the test rate values: 10 mm/min, 100 mm/min, 200 mm/min, 500 mm/min, and 1000 mm/min.

For the thinner composite (with eight layers of quadriaxial glass fiber fabric), the differences are smaller, all in a range of ±10% of the average calculated with the values obtained for each test rate and each characteristic; this leads to a better quality of the thinner composite. For the thicker composite, the only characteristic that overpasses this variation range is the energy at first peak, with the others (including F_max_, Young’s modulus, flexural stress and strain at the first peak, and displacement at the first peak) being in a range of ±7%.

## 4. Failure Mechanisms in Bending the Composite

Peter Beaumont [62] agreed that “a major difficulty in designing composite structures is how to predict damage initiation and damage evolution, and safe operating limits to ensure structural integrity”. Therefore, design of composite structures also relies on understanding the failure mechanisms, especially at lower-scale size. As failure mechanisms of a composite, transverse ply cracks, delamination, and breaks in fibers are mentioned. These are also identified in the post-test SEM images of the formulated composites for this study.

Understanding these failure mechanisms is crucial for designing composite structures that can reliably withstand bending loads. Generally, the failure mechanisms of fiber composites in bending include several modes, such as fiber fracture, matrix cracking, delamination, and fiber–matrix de-bonding [18,63,64,65,66]. Matrix cracks occur when the polymeric matrix undergoes tensile or shear stresses beyond its strength limits, resulting in microscopic cracks that propagate under continued loading, leading to loss of stiffness and strength in the composite structure.

In recent years, the methods of investigating failures in the composites have evolved to ED investigations, including micro-X-ray-computed tomography [67,68,69]. Corroborating this method with other ones, including electron scanning microscopy, the researchers could explain and offer solutions to delay or/and minimize failure of a particular composite.

**Figure 10:**
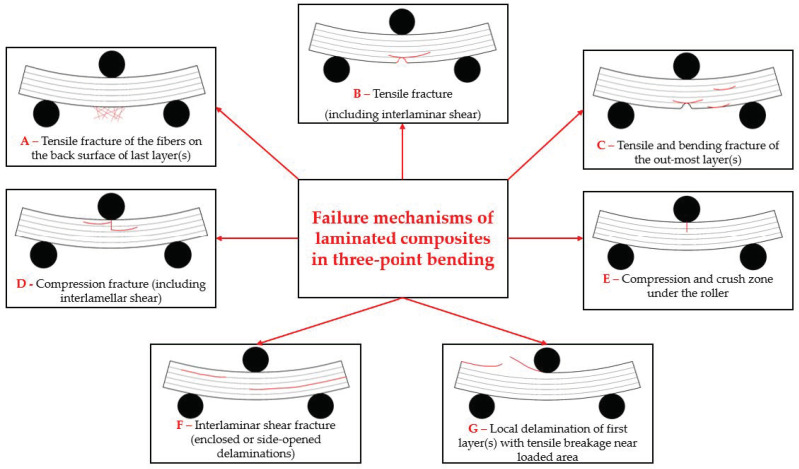
Sketches of failure mechanisms for laminated composites, under three-point bending (based on [70]).

**Figure 11:**
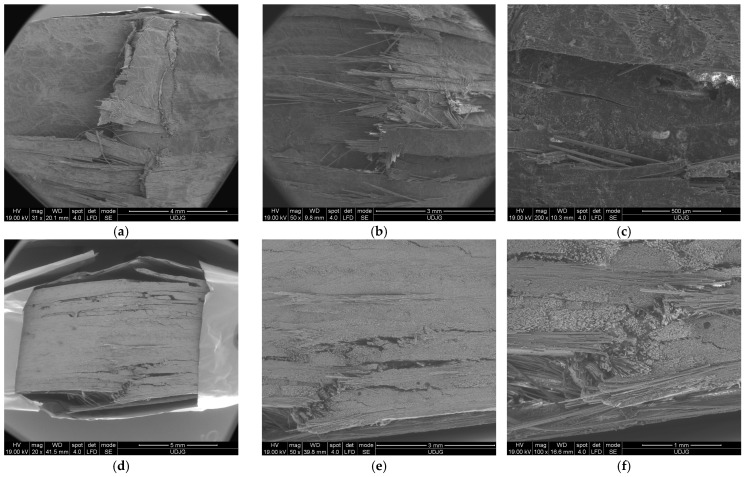
Details of mechanism failure for the composite made of 8 layers of quadriaxial fabric, tested at 10 mm/min: (**a**–**c**) face view and (**d**–**f**) lateral view of the same specimen.

**Figure 12:**
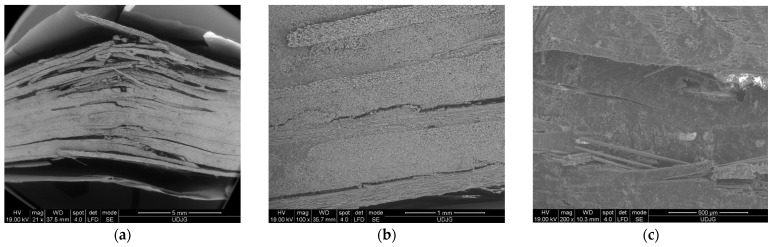
Details of the mechanism of failure for the composite made of 8 layers of quadriaxial fabric, tested at 500 mm/min, lateral view: (**a**) lateral view with alternative delamination between layers and sublayers, with the last layers (in the upper part of the image) being broken; (**b**) detail of the crack tip of a delamination; (**c**) front view of a crack in a subsequent layer, after the first one was broken.

**Figure 13:**
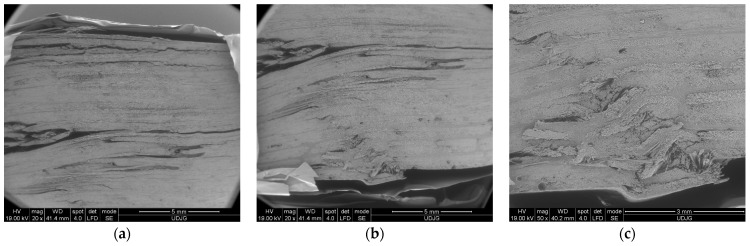
Details of the mechanism of failure for the composite made of 16 layers of quadriaxial fabric, tested at 10 mm/min: (**a**) detail of the lateral face of the composite, where the upper surface was in contact with the roller in the right side; (**b**) lateral view detail from the bottom of the composite; (**c**) detail at a magnification of ×50 of the last layers of the composite.

**Figure 14:**
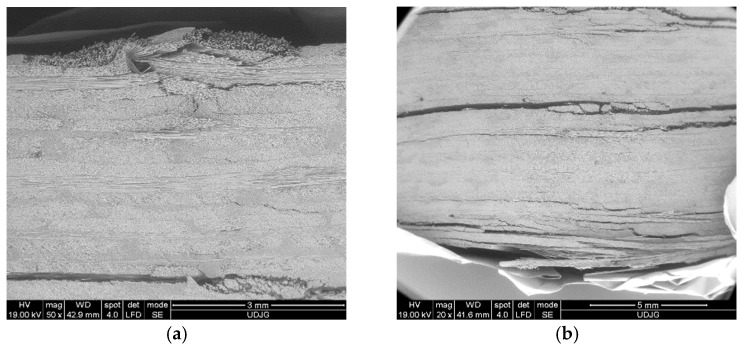
Lateral view of the specimen with 16 layers, at 500 mm/min: (**a**) the upper part of the composite, with damages near the roller contact; (**b**) the bottom part of the composite, with large delamination between last layers and broken layers caused by excessive bending strain.

Sketches of the failure mechanisms characterizing the three-point bending of laminated composites are presented in Figure 10, based on ISO 14125:1998 fiber-reinforced plastic composites [70]. For the determination of the flexural properties, a G-sketch was added for this bending load; the fracture near the acting roller was obtained for all samples, with both 8 layers and 16 layers. These sketches present only one mechanism, but the tested composites present combined failures, resulted from several of the individually described mechanisms. Figure 11, Figure 12, Figure 13 and Figure 14 present SEM images (at small magnifications), where one or more of the failure mechanisms enumerated in Figure 10. Letters added to the SEM image comment refer to the mechanisms in Figure 10.

The micro–mezzo scale of the failure mechanisms includes the failures of yarns and fibers.

When subjected to bending loads, reinforcement fibers within the composite experience tensile stresses. If these stresses exceed the fiber tensile strength, then they may fracture. The fiber fracture reduces the load-carrying capacity of the composite and can lead to catastrophic failure. Delamination refers to the separation of layers within a composite laminate. In bending, interlaminar shear stresses can cause delamination between adjacent layers of fibers, leading to reduced structural integrity and performance. The delamination is more pronounced in zones where flexural stress is higher, usually the first layers, dominated by traction and bending due to the action of the loading body and the last layers where the fiber strains are larger. Delamination in the central part of the composite under bending could be caused by the non-uniform technological application (here, referred to as the lay-up) of the matrix. Fiber–matrix de-bonding occurs when there is a loss of adhesion between the fibers and the matrix. This can happen due to various factors, such as inadequate surface treatment of fibers, environmental degradation, or mechanical loading.

Figure 11 presents failure mechanisms in the composite with eight layers, after being tested at 10 mm/min: (a) front view—the left side shows a crush damage caused by the loading roller (failure mechanism E from Figure 10); following this damage, there is a break of the first layer, where the yarns towards the edges are clearly detached; at the middle of the specimen, this break is not so widespread, and the yarns are not entirely broken (failure mechanism G from Figure 10); they are detached from the subsequent layer.

Figure 11b shows a detail of the broken yarns on the first layer, near the zone in direct contact with the loading roller; (c) shows a detail at a magnification of ×200, highlighting a crack in this layer, which is very probably only along the fibers; several glass fibers are broken and a matrix fragment has detached from the fibers; (d) shows a lateral view of the same specimen; the load direction is from down to up; this SEM image proves a combination of failure mechanisms with different intensities; the last three layers are broken (failure mechanism A from Figure 10); delamination is more visible in the first and last thirds of the specimen’s thickness (failure mechanism F from Figure 10); delamination is visible between layers and also between the sublayers of the pre-peg; (e) shows details of the cracks of the layers near the zone where the roller acts; (failure mechanism F from Figure 10) (f) shows lateral views of the same detail at a magnification of ×200.

Figure 12 presents SEM images of the 8-layer composite, but under a test rate of 500 mm/min: (a) as compared to the same composite under 10 mm/min testing, this presents delamination almost on the entire thickness; delamination does not occur symmetrically, meaning that this damage reduces the loading on the following layer and the separation appears in the other side of load direction (bottom–up); (b) shows a detail of a delamination tip; (c) shows a view of a stopped crack that is visible on the subsequent layer because the yarns on the first layer were broken.

Figure 13 shows SEM images at low magnification (×20 and ×50), revealing delamination (layer or sublayer separations) and separations between yarns and partial breaks of yarns. Large delamination occurrences are found to be concentrated in the opposite part of the composite face where the roller acts.

Figure 14 presents a lateral view of the composite with 16 layers, tested at 500 mm/min. The delamination is greater and the openings between the delaminated layers are larger.

At a micro level, i.e., at the fiber size, the failure mechanisms are qualitatively similar to the results of the tested rates and the thickness of the composite, but the densities of the failures are different.

**Figure 15:**
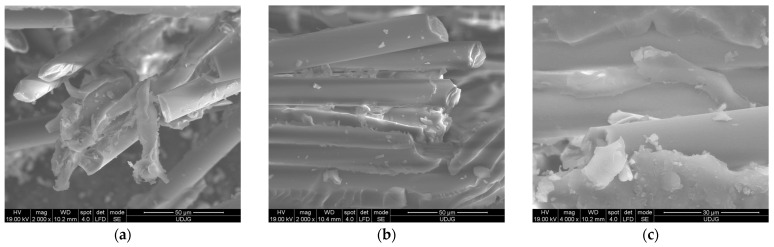
Failure mechanisms at micro level for 8-layer composite, with test rate 10 mm/min, front view (first layer in contact with the loading roller): (**a**) broken glass fibers, matrix de-bonding, fragmentation; (**b**) broken fibers and traces of de-bonded fibers in the matrix; (**c**) a broken fiber still partially attached to the matrix.

Figure 15 shows failures of fibers and matrix, at micro level, for the 8-layer composite, at test rate of 10 mm/min, from the front surface (the first layer in contact with the loading roller): (a) shows fibers broken in tension and bending (with fracture cross-section perpendicular to the fiber length or oblique), matrix de-bonding, fragmentation, and twisting; (b) shows broken fibers and traces of de-bonded fibers in the matrix; the upper fiber was bent and then broken; (c) shows a broken fiber that is still partially attached to the matrix.

**Figure 16:**
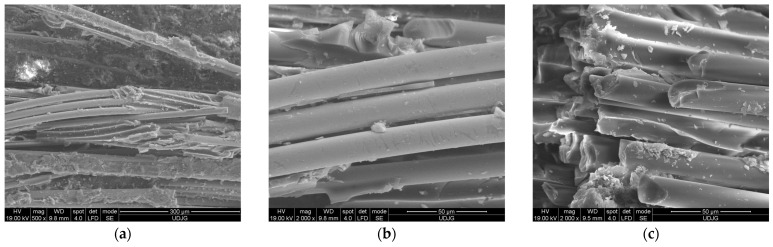
Failure mechanisms at micro level for 8-layer composite, with test rate 500 mm/min, front view (first layer in contact with the loading roller): (**a**) bent fibers (some are already broken); (**b**) broken fiber in bending and the matrix rupture nearby and bent fibers with small fragments of matrix still attached; (**c**) different fractures of the fibers and matrix with traces of the de-bonded fibers.

At a higher test rate, the matrix de-bonding process takes place even if the fibers are not broken (Figure 16a,b). The cross-sections of the broken fibers have a different aspect: the net broken surface that is almost perpendicular to the fiber length or oblique fractures is shown in Figure 16c; the location of this broken yarn is on the top of the composite near the roller contact. One can also see the trace of de-bonded fibers and cracks in the matrix, that remains between the fibers.

## 5. Conclusions

This study pointed out the characteristics of a composite made of quadriaxial glass fiber fabrics and a high-quality bicomponent epoxy resin (Biresin^®^ CR82 and hardener Biresin^®^ CH80-2) by a laboratory technology, including laying-up, pressing, natural aging, and heat treatment. The results obtained by three-point bending testing, for five test rates (10 mm/min to 1000 mm/min), pointed out that, for the same thickness of the composite, the characteristic stresses at the first peak, the flexural modulus, the strain at the first peak, and the energy at the first peak were not sensitive to testing in the rating range of 10–1000 mm/min. The energy at the last peak is double for the 16-layer composite as compared to that of the 8-layer composite, but the specific energy (as the energy in the cross-section area) has close values, 103.47 kJ/m^2^ for the 8-layer composite and 106.51 kJ/m^2^ for the 16-layer composite.

The characteristics clearly influenced by the composite thickness were the recorded maximum force, F_max_, the time to reach this value, t_(Fmax)_, and the displacement to reach the maximum force, d_(Fmax)_. Thus, for the 8-layer composite (with an average thickness of 6.4 mm), F_max_ was around 2000 N, t_(Fmax)_ was longer for a test rate of 10 mm/min (51 s), and d_max_ was 8.70–9 mm; this was insensitive to the change in the test rate. For the 16-layer composite (with an average thickness of 12.48 mm), F_max_ was around 8000 N, t_(Fmax)_ was shorter for test rate 10 mm/min (29.6 s), and d_max_ was around 5 mm; this was also almost insensitive to the test rate.

The mechanical characteristics, such as the Young’s modulus and the flexural stress, showed a slight decrease when their values were compared between the lowest test rate (10 mm/min) and the highest one (1000 mm/min); this was the case for both of the composites. The strain at the first peak showed no clear dependence on the test rate. Thus, for the 8-layer composite, Young’s modulus varies from 20.03 GPa to 23.4 GPa; for the 16-layer composite, this characteristic varies from 17.85 GPa to 19.20 GPa. This means that the thicker composite has this characteristic to a lower degree, with 17.94% (at 10 mm/min), 12.23% (at 100 mm/min), 15.66% (at 200 mm/min), 20.33% (at 500 mm/min), and 10.67% (at 1000 mm/min), respectively, as compared to the values for the 8-layer composite. The same tendency is kept for the flexural stress at the first peak. The 8-layer composite has flexural stress values between 502.84 MPa and 565.47 MPa, and the 16-layer composite has flexural stress values 546.8 MPa and 475.6 MPa; this means that the thicker composite has this characteristic to a lower degree, with 8.66% (at 10 mm/min) and 3.74% (at 1000 mm/min), respectively, as compared to the values for the 8-layer composite. The strain at the first peak varies between 3.32% and 3.63% for the 8-layer composite and between 3.52% and 4.02% for the 16-layer composite. Except for the tests with 1000 mm/min, the values for the thicker composite are a little bit higher than those for the 8-layer composite (not more than 0.6%).

This conclusion could be expressed for another specific energy characteristic: the ration of absorbed energy till F_max_ and the fiber mass for the tested composites. For the composite of 8 layers, this energy at 10 mm/min is 9.01 J, while for 16 layers, it is 18.37 J; the 8-layer composite contains 840 g of glass fibers, while 16-layer composite contains 1675 g of glass fibers. A simple calculus gives the energy absorbed for the fiber mass unit, 10.72 J/kg, and that 10.97 J/kg. This indicates that both of the composites are similar in their properties. This is a good characteristic, meaning that a designer could use the composites with different thicknesses and attain the same value for the specific energy. This is the result of a well-controlled technology (here, at laboratory scale) as the ratio fiber mass/composite mass ratio remains the same for both composites.

The failure mechanisms included delamination, crack propagation between yarns, yarns’ breakage, composite crushing under the loading roller. SEM images reveal that delamination has different aspects, taking into account the composite thickness. The absorbed specific energy, as a ratio of the energy at the peak force and that at the cross-section of the specimens, has similar values. For the 8-layer composite, this characteristic varied between 100.99 kJ/m^2^ and 105.93 kJ/m^2^, with an average of 103.4 kJ/m^2^ and a standard deviation of 1.9 kJ/m^2^, representing 1.88% of the average value. For the 16-layer composite, this characteristic has as extreme values of 93.99 kJ/m^2^ and 121.64 kJ/m^2^; there is a larger range, meaning that the average value for all the test rates is 106.5 kJ/m^2^. There is a larger standard deviation of 11.2 kJ/m^2^; this represents 10.58% of the average value. This could be explained by the fact that the increase in the composite’s thickness can lead to more local defects and non-uniformity in the distribution of the components, which are accumulated during the composite fabrication.

## Figures and Tables

**Table 1:** Characteristics of the plates for cutting the samples.

Composite	Fabric Mass	Plate Mass	Resin Mass *	Fabric/Panel Mass Ratio **	Areal Density ***	Thickness in 4 Points				
						1	2	3	4	Average
	[g]	[g]	[g]		[kg/ m^2^]	[mm]				
0	1	2	3	4	5	6				
8-layer plate	840	1130	290	0.743	12.55	6.41	6.21	6.27	6.70	6.40
16-layer plate	1675.00	2220.00	545.00	0.75	18.61	12.47	12.43	12.51	12.56	12.48

* The resin mass = panel mass–fabrics mass, meaning (column 2–column 1); ** fabric/panel mass ratio = fabric mass/panel mass, meaning (column 1/column 2); *** areal density = panel mass/panel surface (0.09 m^2^).

**Table 2:** Information on energy at first peak and specific energy.

	8 Layers					16 Layers				
Test rate [mm/min]	10	100	200	500	1000	10	100	200	500	1000
Energy at first peak, E [J]	9.01	9.47	9.34	8.77	9.06	18.37	16.82	21.10	18.82	17.79
E_average_, at same thickness, [J]	9.13					18.58				
Standard deviation for E, [J]	0.66	0.77	0.93	0.74	0.66	1.58	2.48	1.86	4.74	1.98
Coefficient of variation, %, for E	7.33	8.13	9.96	8.44	7.28	8.60	14.74	8.81	18.52	11.12
h [mm]	6.34	6.72	6.38	6.04	6.72	12.42	12.91	12.68	12.60	12.64
b [mm]	13.60	13.80	13.82	13.98	13.35	13.64	13.45	13.68	13.38	14.48
Specific energy, E_s_ [kJ/m^2^]	104.47	102.11	105.93	103.86	100.99	108.43	96.86	121.64	111.63	93.99
E_s average_, at same thickness, [kJ/m^2^]	103.47					106.51				

The coefficient of variation [%] of a set of values for a characteristic is calculated as the ratio of standard deviation and average value of the characteristic, expressed in percentage. In Table 2, the coefficient of variation, as percentage, is calculated for energy absorbed at first peak of the recorded force, F_max_.

## Data Availability

Data are contained within this article.
